# Supplementary material for: The Prognostic Value of Pain Phenotyping in Relation to Treatment Outcomes in Patients with Axial Spondyloarthritis Treated in Clinical Practice: A Prospective Cohort Study
Source: J Clin Med. 2021 Apr 2;10(7):1469. doi: 10.3390/jcm10071469 (PMC8038186; doi:10.3390/jcm10071469)
Supplement: Supplementary file 1 [file jcm-10-01469-s001.pdf]

Journal of
Clinical Medicine

MDPI

Supplementary Material

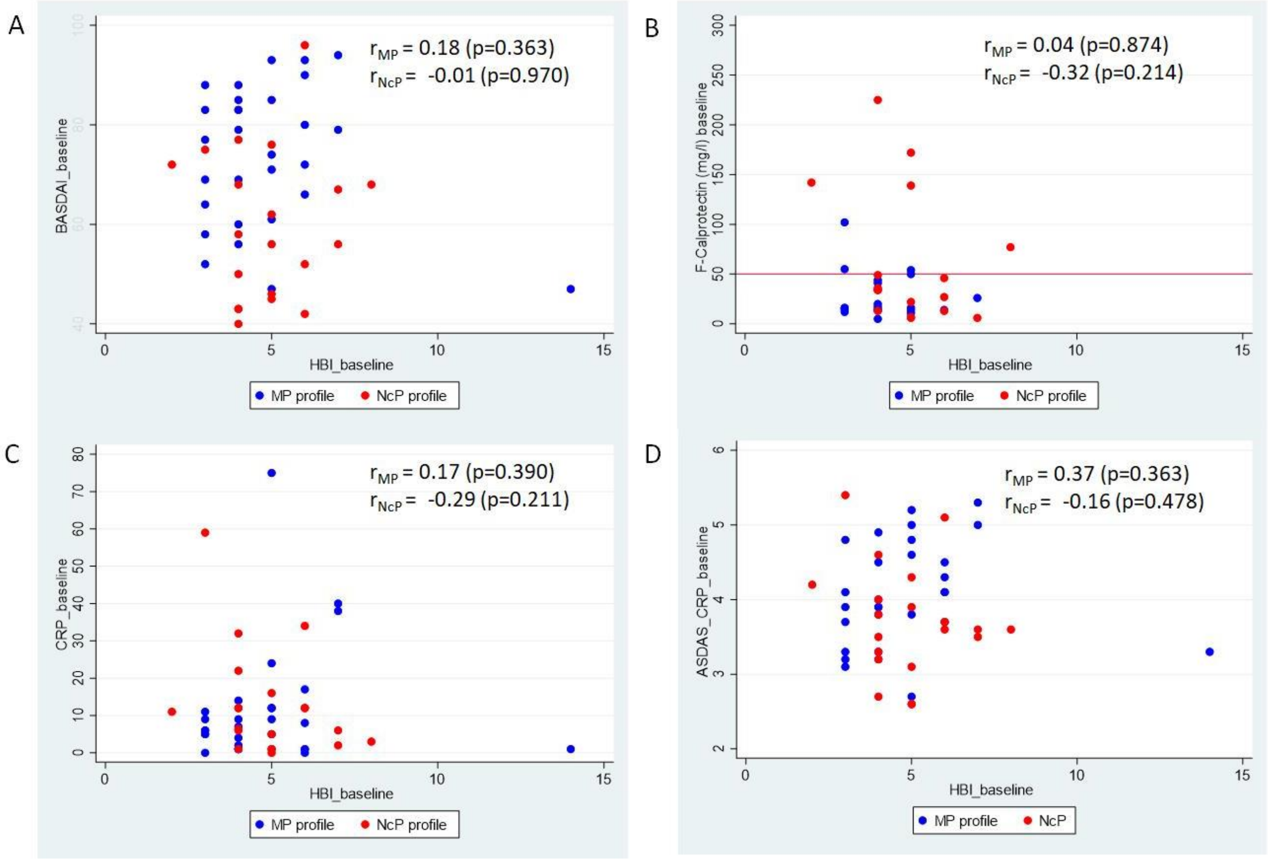

Figure S1. Scatter diagrams of spearman correlation between Harvey Bradshaw Index (HBI) and variables reflecting disease activity.

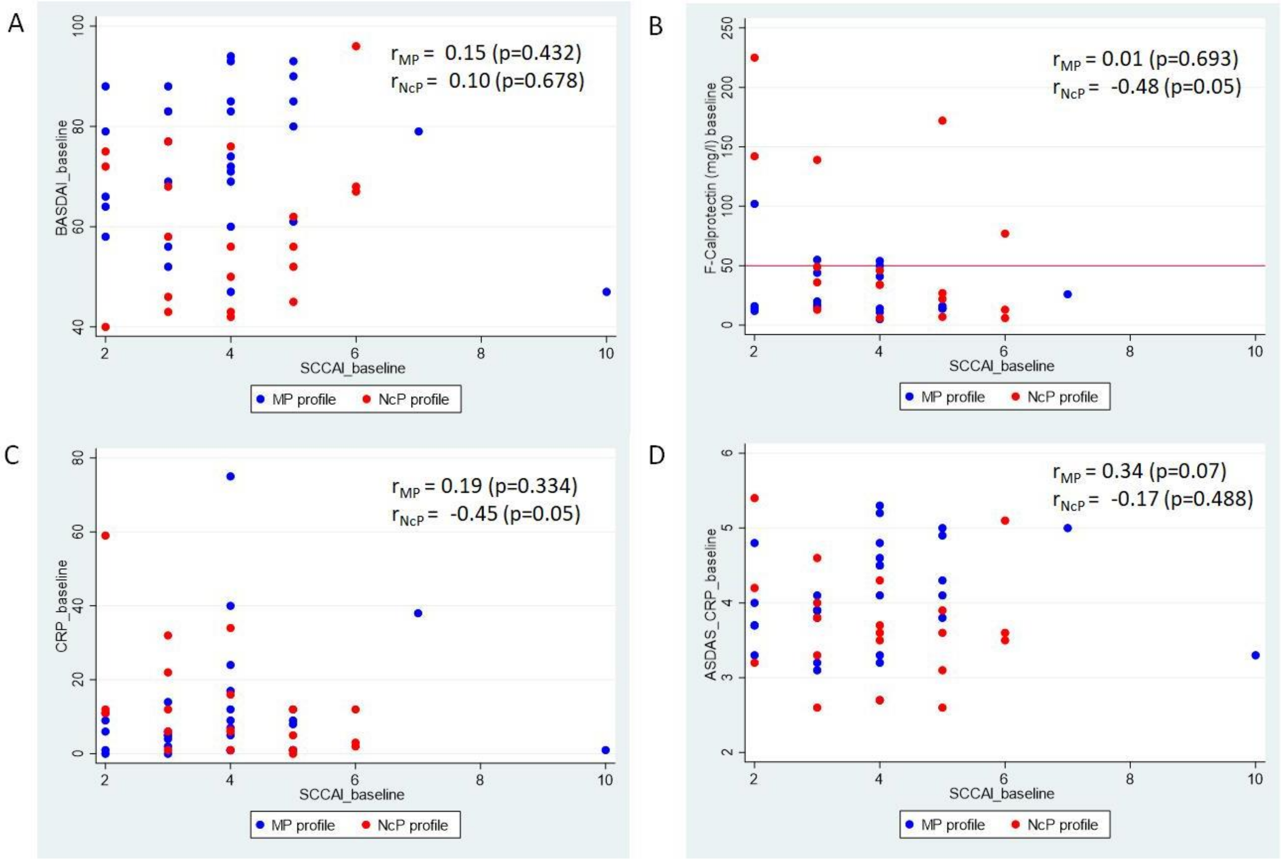

Figure S2. Scatter diagrams of spearman correlation between Simple Clinical Colitis Activity Index (SCCAI) and variables reflecting disease activity.
